# Outcome of a Low-Cost Glaucoma Implant versus the Baerveldt Glaucoma Implant for Paediatric Glaucoma in a Tertiary Hospital in Egypt

**DOI:** 10.1155/2019/5134190

**Published:** 2019-12-20

**Authors:** Mahmoud F. Rateb, Hazem Abdel Motaal, Mohamed Shehata, Mohamed Anwar, Dalia Tohamy, Mohamed G. A. Saleh

**Affiliations:** Department of Ophthalmology, Faculty of Medicine, Assiut University, Asyut, Egypt

## Abstract

**Purpose:**

To compare safety and efficacy between a low-cost glaucoma drainage device (GDD), the Aurolab aqueous drainage implant (AADI), and the Baerveldt glaucoma implant (BGI) in refractory childhood glaucoma in Egypt.

**Methods:**

This is a retrospective study of patients who received either an AADI or BGI at a tertiary care postgraduate teaching institute. Children aged <16 years with uncontrolled intraocular pressure (IOP) with or without prior failed trabeculectomy who completed a minimum 6-month follow-up were included. The outcome measures were IOP reduction from preoperative values and postoperative complications.

**Results:**

Charts of 57 children (younger than 16 years old) diagnosed with refractory childhood glaucoma were included. Of these, 27 eyes received AADI implants (group A), while 30 received BGI implants (group B). The mean preoperative baseline IOP was 34 ± 5 mmHg in group A versus 29 ± 2 mmHg in group B (*p*=0.78) in patients on maximum allowed glaucoma medications. In group A versus group B, the mean IOP decreased to 13.25 ± 8.74 mmHg (*p*=0.6), 12.8 ± 5.4 mmHg (*p*=0.7), and 12.6 ± 5.6 mmHg (*p*=0.9) after 1 week, 3 months, and 6 months, respectively. However, in group A, an anterior chamber reaction appeared around the tube in 14 cases starting from the first month and resolved with treatment in only 4 cases. In the other 10 cases, the reaction became more severe and required surgical intervention. This complication was not observed in any eye in group B.

**Conclusion:**

AADI, a low-cost glaucoma implant, is effective in lowering IOP in patients with recalcitrant paediatric glaucoma. However, an intense inflammatory reaction with serious consequences developed in some of our patients; we believe these events are related to the valve material. We therefore strongly recommend against its use in children.

## 1. Introduction

Childhood glaucoma is a blinding disease with a prevalence of 0.1/1000 to 1.1/1000 in children in different parts of the world, but a remarkably higher prevalence in developing countries (up to 0.051%) [1, 2]. Glaucoma drainage devices (GDDs), such as the Ahmed glaucoma valve (AGV) or Baerveldt glaucoma implant (BGI), have been reported to have high success rates in the management of childhood glaucoma when used in either a primary surgical procedure or following other angle-based glaucoma surgeries, such as trabeculectomy [3]. Furthermore, 5-year results of the tube versus trabeculectomy (TVT) study found BGI implantation had a higher success rate and lower rate of reoperation than trabeculectomy, with a similar intraocular pressure (IOP) reduction and need for glaucoma medications [4].

The implantation BGI represents a substantial economic burden to a large section of the population in developing countries. However, a low-cost prototype of the Baerveldt implant that has recently become available called the Aurolab aqueous drainage implant (AADI, Aurolabs, Madurai, India). Its low cost, at approximately 1/15, the price of the BGI is a very strong advantage, and the efficacy and safety of AADI implant was comparable with BGI in recent studies [5, 6], but little data are available regarding the safety and efficacy of the AADI outside India, the manufacturing country of the device.

## 2. Aim of the Work

To compare safety and efficacy between a low-cost glaucoma drainage device (GDD), the Aurolab aqueous drainage implant (AADI), and the Baerveldt glaucoma implant (BGI) in refractory childhood glaucoma.

## 3. Patients and Methods

### 3.1. Study Design

This is a retrospective study of children who had either AADI implants or BGI implants who were included in group A and B, respectively.

### 3.2. Study Venue

Surgeries were performed at Assiut University Hospital, a tertiary-level care institute in Egypt, between April 2016 and May 2018 with a minimum of 6 months of follow-up were included.

### 3.3. Inclusion Criteria

The inclusion criteria were as follows:

- Age <16 years old
- Eyes with uncontrolled IOP refractory to medical treatment
- Eyes considered at high risk of failure/complications following conventional filtering surgery, such as those with excessive conjunctival scarring after prior ocular surgery or extremely thin sclera in buphthalmos
- A minimum of 6 months of postoperative follow-up

### 3.4. Exclusion Criteria

The exclusion criteria were as follows:

- Corneal abnormalities that could lead to erroneous IOP readings. However, corneal haze due to buphthalmos or high IOP was not considered an exclusion criterion.
- Uncontrolled systemic diseases such as congenital cardiac abnormalities, uncontrolled seizure disorders, or any other conditions rendering the child unfit for general anaesthesia.
- Glaucoma secondary to uveitis even if the uveitis was controlled before surgery so that any postoperative reaction could be solely attributed to the procedure and not any underlying inflammatory disease.
- Any other active ocular diseases (e.g., ocular infection).

## 4. Surgical Procedure

As a standardized procedure, all surgeries were performed by one experienced surgeon (M. R.) following the same technique for Baerveldt or AADI implantation: A fornix-based conjunctival opening is created in the superotemporal quadrant. The AADI tube is checked for patency and is ligated with a 6–0 vicryl (braided coated Polyglycan by Ethicon, Johnson & Johnson, USA) suture, and occlusion is tested. Venting incisions are made anterior to the ligated tube, approximately 3-4 pairs, and vents are checked for patency. Adjacent recti are identified and hooked, and the underbelly was cleaned, prior to placement of the wings of the AADI or BGI underneath them. The implant plate is fixed with two interrupted 9–0 prolene suture (monofilament polypropylene blue; Ethicon, Johnson & Johnson, FSSB NADELN GMBH, Germany) with the anterior edge of the plate approximately 10 mm posterior to the limbus. The suture knots are rotated into the fixation eyelets. The tube length is shortened to approximately 3 mm with a bevelled tip opening towards the cornea. A 23-gauge needle is used to create a track 2 mm behind the limbus through which the tube is inserted into the anterior chamber just anterior and parallel to the iris for anterior chamber placement and behind the iris for sulcus placement. The tube is inserted through the needle track and secured to the sclera with a figure-of-eight 10–0 nylon suture (monofilament polyamide black, Ethilon; Ethicon, Johnson & Johnson, USA). Almost the entire length of the tube is covered with a corneal patch graft or a partial thickness sclear flap if graft is not available. The conjunctiva and Tenon are brought forward and secured back into position with 8–0 vicryl (braided coated polyglactin 910 violet; FSSB NALDEN GMBH, Germany) wing and continuous sutures. At the end of the procedure, antibiotic and steroid eye drops and ointment are applied to lower fornix. Mitomycin or other antimetabolites have never been used.

### 4.1. Outcome Measures

The primary outcome measure was IOP reduction relative to preoperative values, and the secondary outcome measure was the occurrence of postoperative complications.

### 4.2. Statistical Analysis

Data were recorded in the form of excel spreadsheets. Statistical analysis was performed using SPSS program, version 21. Mann–Whitney test was used to compare means between both study groups. Wilcoxon signed rank test was used to compare means across different study time points within the same study group.

## 5. Results

Charts of 57 children (33 males and 24 females) who met the inclusion criteria were included in the study.

Group A included twenty-seven eyes that received an AADI implantation (in the primary procedure in 12 eyes; the remaining 15 eyes had one or more previously failed glaucoma surgeries). Group B included 30 eyes that received a BGI. The BGI was implanted as a primary procedure in 14 eyes and as a second surgery in 16 eyes. The baseline characteristics of both study groups are shown in Table 1.

### 5.1. Primary Outcome Measure

Among patients on the maximum allowed glaucoma medications, the mean preoperative baseline IOP was 34 ± 5 in group A and 29 ± 2 mmHg in group B. Following surgery, IOP was lower in both groups at 1 week and 3 and 6 months postoperative. The change of IOP from baseline was statistically significant (*p* < 0.05) at all time intervals, although the difference between the study groups remained statistically insignificant.

Table 2 summarizes the mean IOP values recorded in both study groups at different time points and their *p* values.

### 5.2. Secondary Outcome Measures

In group (A) patients, adverse reactions included an intense anterior chamber reaction (ranging from cells and flare to severe fibrinous reaction) and occurred in 14 cases. Onset ranged from the first to the third month postoperative but peaked in the third month.

**Figure 1:**
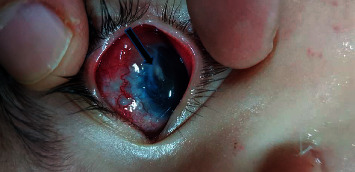
An arrow pointing to a dense fibrous reaction around the tube causing IOP to increase above target levels.

**Figure 2:**
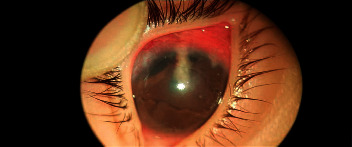
A dense fibrous sleeve formed around the tube, similar to the case shown in Figure 1.

**Figure 3:**
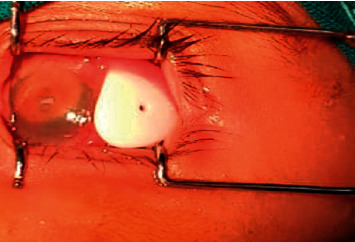
Extrusion of the valve due to severe erosive conjunctivitis.

**Figure 4:**
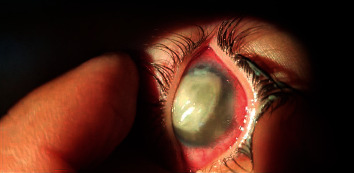
Intense corneal infiltrate and AC reaction.

The reaction typically started around the site of tube entrance, with flare and dense fibrinoid reactions around the tube. This reaction was successfully controlled by increasing the dose of topical prednisolone and cycloplegics in only 4 eyes (Figures 1 and 2). In the other 10 cases, the fibrinous reaction required surgical intervention in the form of AC wash and dissection of the membranes from the iris and tube, synecholysis, and pupilloplasty. The fate of this intense reaction was as follows:

1. Tube occlusion in 3 eyes resulted in re-elevation of the IOP to levels above the target IOP. Two of those eyes required reimplantation of another GDD. The mean time for reintervention was 5.8 months after surgery.
2. Two other eyes developed significant cataracts that were removed at the age of 12 and 13 months with IOL implantation (9 months and 11 months after AADI implantation surgery, respectively).
3. Two eyes developed severe conjunctivitis that required intensifying treatment. These two cases progressed to spontaneous erosion and extrusion of the valve within 2-3 weeks (Figure 3).
4. In two eyes, the reaction extended to endophthalmitis. In one eye, this was managed by pars plan vitrectomy. Vitreous samples showed no growth of microorganisms. The other eye developed resistant corneal abscesses that were negative on culture for microorganisms, and a corneal graft was immediately applied to salvage the patient's vision (Figure 4).
5. One eye developed occlusio pupillae that required surgical synecholysis.

**Figure 5:**
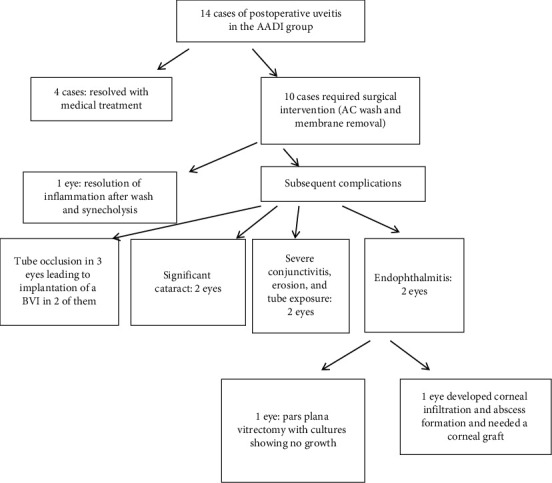
Flowchart summarizing the fate of the postoperative reaction noted in some of the study subjects.

Other complications included fibrous encapsulation around the tube plate in one eye and severe corneal oedema in one eye (oedema not related to the location of the tube) that developed severe uveitis and IOP elevation (this involved the lower half of the cornea and was therefore not due to the tube touching the endothelium). These complications are summarized in Table 3 and Figure 5.

Secondary outcomes in group B patients were similar to the usual complications reported in the literature after BGI and included prolonged hypotony after suture release in 2 eyes, late-onset cataract necessitating surgical intervention in 2 eyes, and encapsulation and raised IOP in one eye.

## 6. Discussion

Childhood glaucoma is classified into different entities with diverse aetiologies and prognoses, including primary congenital glaucoma and secondary glaucoma associated with other ocular or systemic diseases [7]. Filtering procedures such as trabeculotomy and trabeculectomy augmented with antifibrotic agents represent the most commonly performed procedure for children. Because of the higher risk of complications associated with exposure to antifibrotic agents in children, there has been a paradigm shift towards the implantation of GDDs either primarily as well as in resistant cases [8].

Worldwide, the two most commonly used GDDs are the AGV and the BGI. The AGV has a built-in valve that prevents hypotony in the early postoperative period. In contrast, the BGI is nonvalved and requires the tube to be temporarily ligated to prevent early postoperative filtration until there is adequate encapsulation around the endplate to regulate flow after removal of the ligature (approximately 5-6 weeks) [9]. Data pooled from randomized controlled studies that compared the AGV with the BGI at the end of 5 years showed that the mean IOP was lower in patients on fewer medications in the BGI group than in the AGV group possibly because the larger surface area and less encapsulation rate of the BGI [10]. Although no previously published study has compared the BGD 350 with the AADI (which also has a surface area of 350 mm^2^), it is presumed that these two GDDs would be equivalent as the former was the design inspiration for the latter. Compared with Ahmed valve (AGV), AADI showed superiority in success rates (90% with AADI compared with 80% with AGV) which were statistically significant [6]. Kaushik et al. reported that the efficacy of AADI at one year was more than 90%, which dropped to 82% at two years. This is similar to that reported in previous studies using the BGI in children. There were no serious complications or endophthalmitis during the follow-up period [5]. In our study, the AADI yielded similar provisional success rates in many cases and a mean IOP reduction similar to those reported in previous studies that evaluated the use of the AADI. However, approximately 48% of the eyes in our series experienced intense fibrinous postoperative reactions that required intensifying medical treatment, and some cases required a second surgical intervention to clear the AC of fibrin and exudates. Some of the cases ended with either failure of the valve or loss of vision due to endophthalmitis or corneal ulcers. Those events, if unrecognized and undertreated, might jeopardize surgery outcomes and even put the eye at other risks, such as phthisis bulbi, especially in those children whose eyes are at high risk of severe inflammatory reactions [11].

In settings other than tertiary centres, in which repeated examinations of patients under general anaesthesia are feasible, and this complication and its devastating consequences could be easily missed. The alarming rate of postoperative reactions reported in our series has not been described in previous series. We strongly propose that the silicon material used in the valve may be the reason for these reactions as we did not encounter any similar reaction when using the Ahmed valve or Baerveldt glaucoma implant.

## 7. Conclusion

The AADI, a low-cost GDD, is effective in lowering IOP. However, in our patients, it resulted in serious complications we believe were related to the material contained in the valve. We strongly recommend against its use in children. In the future, randomized prospective comparative studies are needed to validate the results of our study.

## Figures and Tables

**Table 1:** Baseline characteristics of the study subjects.

	Group A, *N* = 27	Group B, *N* = 30
Age (months)	28.12	34.24
Sex		
Male	17	16
Female	10	14
Glaucoma diagnosis		
Primary congenital glaucoma	19	22
Aphakic glaucoma	2	3
Pseudophakic glaucoma	2	2
Traumatic glaucoma	1	0
Glaucoma in a vitrectomized eye	1	2
Sturge–Weber syndrome	1	0
Axenfield–Rieger syndrome	1	1
Mean preoperative IOP (mmHg)	34 ± 5	29 ± 2.2
Mean preoperative antiglaucoma medication	3.2 + 0.6	3.1

**Table 2:** Mean IOP at baseline and different study time points.

	Baseline mean IOP	Mean IOP at week 1	Mean IOP at month 3	Mean IOP at month 6
Group A	34	13.28	12.8	12.6
Group B	29	14.6	14.8	14.9
*p* value	—	<0.001	<0.001	<0.001

**Table 3:** Complications seen in eyes which received AADI implant.

Complication	Number of eyes	Fate
Intense anterior chamber inflammation	14	(i) 4 eyes: resolved with treatment
		(ii) 1 eye: resolved after AC wash
		(iii) 9 eyes: developed subsequent complications
Tube occlusion	3	Implantation of BVI in 2 eyes
Significant cataract	2	
Erosive conjunctivitis with tube exposure	2	
Endophthalmitis	2	(i) 1 eye: resolved with vitrectomy, sterile
		(ii) 1 eye: subsequently developed infectious keratitis necessitating keratoplasty

## Data Availability

Data related to the study subjects are stored in a deidentified manner in a data repository and are available upon request.
