# Corrigendum to “Outcome of a Low-Cost Glaucoma Implant versus the Baerveldt Glaucoma Implant for Paediatric Glaucoma in a Tertiary Hospital in Egypt”

**DOI:** 10.1155/2022/9834763

**Published:** 2022-03-21

**Authors:** Mahmoud F. Rateb, Hazem Abdel Motaal, Mohamed Shehata, Mohamed Anwar, Dalia Tohamy, Mohamed G. A. Saleh

**Affiliations:** Department of Ophthalmology, Faculty of Medicine, Assiut University, Asyut, Egypt

The article titled “Outcome of a Low-Cost Glaucoma Implant versus the Baerveldt Glaucoma Implant for Paediatric Glaucoma in a Tertiary Hospital in Egypt” [1] has been updated to remove a statement in the Discussion section referring to a response received from the manufacturing company as this could not be verified.
